# Re-enforcing the strategy of targeting MEK/ERK signaling to overcome acquired resistance to third generation EGFR inhibitors

**DOI:** 10.18632/oncoscience.539

**Published:** 2021-08-04

**Authors:** Wen Zhao, Shi-Yong Sun

**Affiliations:** ^1^Department of Oncology, The First Affiliated Hospital of Xi’an Jiaotong University, Xi’an, Shaanxi, People’s Republic of China; ^2^Department of Hematology and Medical Oncology, Emory University School of Medicine and Winship Cancer Institute, Atlanta, GA 30322, USA

**Keywords:** lung cancer, osimertinib, MEK/ERK, acquired resistance

Lung cancer, which consists of over 80% non-small cell lung cancer (NSCLC), is the leading cause of cancer-related death in the world [1]. The discovery of the connection between mutant epidermal growth factor receptor (EGFR) and cancer cell response to EGFR-tyrosine kinase inhibitors (EGFR-TKIs) that led to development of EGFR-targeted therapy has revolutionized the treatment of NSCLC. During the last two decades, EGFR-TKIs have advanced from 1st and 2nd generation to 3rd generation agents represented by osimertinib (AZD9291). Because of its promising performance in clinical trials, osimertinib has been approved to treat NSCLC patients with *EGFR* activating mutations (e.g., exon 19 deletion and exon 21 point mutation L858R) as a frontline therapy or those progressed from 1st or 2nd generation EGFR-TKIs due to *EGFR* T790M mutation as a second-line treatment option [2, 3].

Similar to 1st and 2nd generation EGFR-TKIs, patients still inevitably develop acquired resistance to 3rd generation EGFR-TKIs, limiting their long-term efficacies. The most commonly known mechanism is the acquisition of novel *EGFR* resistance mutations, such as the C797S mutation. Additionally, some EGFR-independent mechanisms, such as amplification of the *MET* gene, also play important roles. RAS/RAF/MEK/ERK signaling plays a critical role in many biological outputs, such as proliferation, survival, differentiation and apoptosis. Upregulation of the RAS/RAF/MEK/ERK signaling pathway has also been suggested to contribute to acquired resistance to 3rd generation EGFR-TKIs; this can occur due to alteration of upstream membrane receptor proteins such as MET and HER2 caused by gene amplification or mutations or alterations directly in the RAS/RAF/MEK/ERK axis including *KRAS* G12S, *KRAS* Q61K, and *BRAF* V600E mutations. These findings strongly suggest a rationale for targeting this signaling axis to overcome acquired resistance to 3rd generation EGFR-TKIs [4].

Our early work shows that osimertinib exerts its inhibitory effects against the growth of EGFR-mutant NSCLC cells primarily through inducing apoptosis; this involves upregulation of Bim and downregulation of Mcl-1 by the modulation of MEK-dependent protein degradation [5]. However, this modulatory activity was lost in cells resistant to osimertinib. Importantly, MEK inhibition with different MEK small molecule inhibitors that decreased Mcl-1 with elevation of Bim, restored the sensitivity of osimertinib-resistant cells and xenografts to osimertinib both *in vitro* and *in vivo*, suggesting an effective strategy for overcoming osimertinib acquired resistance through targeting MEK. As ERK1/2 is a direct downstream kinase of MEK and some promising ERK1/2 inhibitors are undergoing testing in clinical trials, the reasonable question is whether ERK1/2 inhibition can exert activity similar to MEK inhibition in overcoming osimertinib acquired resistance. The recent study by Li et al. [6] addresses this question. In this study, ERK1/2 inhibition, achieved by different ERK1/2 inhibitors including ravoxertinib (GDC0994) and ulixertinib (BVD-523 or VRT752271), combined with osimertinib synergistically decreased the survival of osimertinib-resistant NSCLC cell lines and effectively inhibited the growth of osimertinib-resistant xenografts in nude mice. As with MEK inhibition, ERK1/2 inhibition elevated Bim and decreased Mcl-1; these effects could be further augmented when combined with osimertinib. As speculated, Bim knockout or Mcl-1 overexpression in these resistant cell lines conferred resistance to the combination of osimertinib with an ERK1/2 inhibitor, furthering the notion that modulation of Bim and Mcl-1 critically determines the responses of EGFR-mutant NSCLC cells to osimertinib.

In this study, several osimertinib-resistant cell lines were clearly resistant to both 1st and 2nd generation EGFR-TKIs as well as other 3rd generation EGFR-TKIs, indicating cross-resistance. Interestingly, in the presence of a MEK or ERK1/2 inhibitor, both 1st and 2nd generation EGFR-TKIs became highly effective in significantly decreasing the survival and enhancing apoptosis of osimertinib-resistant cell lines and in inhibiting the growth of osimertinib-resistant xenografts *in vivo*. This finding may have an important clinical implication since it suggests that EGFR-mutant NSCLCs with acquired resistance to osimertinib or other 3rd generation EGFR-TKIs can be treated with either 1st or 2nd generation EGFR-TKIs when combined with an MEK or ERK1/2 inhibitor.

The findings in this study reinforce the significance of targeting the MEK/ERK signaling to prolong the benefit of osimertinib through overcoming acquired resistance to osimertinib. Further investigations of this combinatorial strategy are warranted in the clinic. A recent phase Ib clinical trial called TATTON study (NCT02143466) has actually progressed in this direction [7]. At date cut-off, 36 patients received osimertinib 80 mg orally (p.o.) once a day combined with the MEK inhibitor, selumetinib (25-75 mg, p.o. twice a day; continuous or intermittent). These patients with advanced EGFR-mutant NSCLC had all relapsed from a prior EGFR-TKI treatment. The objective response rate was 42% (95% confidence interval 26% to 59%) and the median duration of response was 16.6 months (95% confidence interval 11.1, not calculable). Moreover, the side effects were tolerable. This study demonstrates the feasibility of combining osimertinib with selumetinib at identified tolerable, active doses. However, only 5 of the 36 patients had prior 3rd generation EGFR-TKI treatment history. Thus, more attention should be paid to the effect of osimertinib combined with MEK/ERK inhibition in patients relapsed from 3rd generation EGFR-TKIs.
